# Comparable efficacy of generic and original alginate for symptom control in PPI-refractory GERD

**DOI:** 10.1038/s41598-025-13400-w

**Published:** 2025-08-04

**Authors:** Kawin Tangvoraphonkchai, Witsarut Manasirisuk, Tanya Apichatvullop, Supatra Srikhajonjit, Manoon Mitpracha, Pattarakorn Promsen, Nutchanun Preechakawin, Khaimook Onthaisong

**Affiliations:** 1 Division of Gastroenterology and Hepatology, Department of Medicine, Faculty of Medicine, Srinagarind Hospital, Khon Kaen University, Khon Kaen, Thailand; 2 Department of Medicine, Khon Kaen Hospital, Khon Kaen, Thailand; 3 Division of Gastroenterology and Hepatology, Department of Medicine, Kalasin Hospital, Kalasin, Thailand; 4 Division of Gastroenterology and Hepatology, Department of Medicine, Pathum Thani Hospital, Pathum Thani, Thailand; 5 Nursing Department, Faculty of Medicine, Srinagarind Hospital, Khon Kaen University, Khon Kaen, Thailand

**Keywords:** Gastroesophageal reflux disease, Generic alginate, Original alginate, Proton pump inhibitors, Reflux disease questionnaire, Gastroenterology, Gastro-oesophageal reflux disease

## Abstract

Gastroesophageal reflux disease (GERD) is a prevalent global condition, affecting 18.1–27.8% of North Americans and 6.3–18.3% of the Thai population. While proton pump inhibitors (PPIs) are the first-line treatment, only about one-third of patients achieve adequate symptom control. Alginate-based medications in combination with PPIs have shown promise, but the comparative effectiveness of generic versus original alginates remains unexplored. To compare the effectiveness of generic alginate (ONE GERD) versus original alginate (Gaviscon Dual Action Suspension) in combination with PPIs for treating GERD symptoms in patients who failed standard PPI therapy.This multicenter prospective randomized controlled non-inferiority trial included 48 patients who failed standard-dose PPI treatment. Patients were randomized to receive either generic or original alginate four times daily for 28 days. Treatment response was evaluated using the Reflux Disease Questionnaire (RDQ) at days 7 and 28. At day 7, both groups showed identical response rates of 45.83%. By day 28, response rates increased to 54.17% for generic alginate and 70.83% for original alginate (*p* = 0.23). Total RDQ scores and symptom-free rates showed no significant differences between groups at both time points. Adverse event rates were comparable (16.67% vs. 8.33%, *p* = 0.66). Analysis of specific symptoms (heartburn, chest pain, and regurgitation) revealed similar improvements in both groups throughout the study period. This study provides evidence supporting the therapeutic equivalence of the generic alginate (ONE GERD) to the original formulation (Gaviscon Dual Action Suspension) in treating symptoms for patients with GERD who have failed PPI therapy. Crucially, the comparable efficacy and safety, coupled with the inherent lower cost of generic medications, suggest significant economic benefits and the potential for wider patient access to effective GERD management. This makes generic alginate a viable and attractive alternative in clinical practice, particularly in resource-limited settings or for patients facing financial constraints, thereby contributing to more equitable healthcare solutions without compromising therapeutic outcomes.

## Introduction

Gastroesophageal reflux disease (GERD) is a common condition worldwide, with prevalence rates in North America, Europe, East Asia, Central Asia, Australia, and South America ranging from 18.1 to 27.8%, 8.8 to 25.9%, 2.5 to 7.8%, 8.7 to 33.1%, 11.6%, and 23.0%, respectively^1^. In Thailand, data indicate that the incidence has approximately doubled over the past five years^2^, reaching as high as 6.3–18.3%^3^. Patients with GERD often present typical symptoms, including heartburn and acid regurgitation, reported at 82.4% and 58.8%, respectively^2^. A study by Treeprasertsuk et al. (2013) found that among a sample population of 2488 individuals in countries selected through random sampling, 855 individuals (34.4%) exhibited specific symptoms of GERD^4^. Currently, international guidelines recommend treating GERD patients with medications, starting with proton pump inhibitors (PPIs)^2^. However, a study by Manabe et al.^5^ showed that, even with PPIs, only 25.7% of GERD patients could control their symptoms in 7 days, with only 35.9% remaining free from heartburn over 28 days. GERD patients often experience chronic symptoms that significantly impact their quality of life compared to healthy individuals, affecting sleep, decreasing vitality, increasing bodily pain, diminishing sexual feelings, and heightening anxiety^6^.

Alginate-based medications offer a distinct therapeutic approach, particularly valuable for patients with GERD refractory to PPIs. The primary physiological mechanism by which alginates exert their therapeutic effects involves a unique interaction with gastric acid. Upon contact with the acidic environment of the stomach, alginates (typically sodium alginate derived from brown seaweed) precipitate to form a viscous, buoyant gel, often referred to as a “raft.” This raft formation is frequently enhanced by the co-formulation with antacids such as calcium carbonate or sodium bicarbonate; these antacids not only help neutralize gastric acid but also react to produce carbon dioxide gas, which becomes entrapped within the alginate gel, increasing its buoyancy and stability. This raft then floats on top of the stomach contents, creating a physical barrier that mechanically impedes the reflux of gastric contents (including acid, pepsin, and bile) into the esophagus. This barrier protects the esophageal mucosa from irritants, thereby alleviating symptoms like heartburn and regurgitation without systemically altering gastric acid production. This physical barrier mechanism is particularly valuable in PPI-refractory GERD because, while PPIs effectively reduce gastric acid secretion, they do not prevent the reflux of non-acidic or weakly acidic gastric contents (such as pepsin and bile salts), nor do they fully address issues like the postprandial “acid pocket” a layer of unbuffered acid that can accumulate near the gastroesophageal junction after meals. Alginates, by forming a raft, can directly target this acid pocket and provide a barrier against these diverse reflux components, which are often implicated in persistent symptoms despite adequate acid suppression.

Currently, various recommendations suggest treating patients with GERD who do not respond to proton pump inhibitors (PPIs) by adding alginate-based medications to help manage their symptoms. Data indicates that the use of original alginate in conjunction with PPIs can control symptoms effectively, with results showing a success rate of up to 56.7% in the first 7 days, and an increase in the percentage of GERD patients free from heartburn over 28 days rising to 54.9%^5^. This approach has been shown to improve patients’ quality of life.

A new class of alginate medications, known as generic alginates, contains similar active ingredients to original alginates but are available at a lower cost, thus reducing treatment expenses for GERD patients. This potential for cost reduction is significant, as it could enhance the affordability and accessibility of effective GERD management, particularly in resource-constrained settings or for patients facing financial barriers to treatment. However, while the principal active ingredient (alginate) may be the same or similar, potential differences between generic and original formulations could influence therapeutic efficacy. These differences might arise from variations in the source and grade of alginate used, the precise quantities of active components (including alginate and any accompanying antacids), and the nature and concentration of excipients. Excipients can affect the physicochemical properties of the alginate raft, such as its viscosity, strength, speed of formation, cohesiveness, and mucoadhesive capabilities. For instance, variations in gelling agents, buffering capacity, or even flavoring agents could subtly alter raft characteristics and, consequently, its effectiveness in forming a robust and persistent barrier. Therefore, even with ostensibly similar active ingredients, the overall formulation plays a critical role in the in-vivo performance of the alginate raft. there have been no studies assessing whether the efficacy of generic alginates matches that of original alginates. This gap in research prompted the current study, which aims to compare the effectiveness of the generic alginate, using ONE GERD (Supplement 1), against the original alginate, using Gaviscon Dual Action Suspension (Supplement 2). Gaviscon Dual Action Suspension is regarded as the original product in this comparison due to its status as the innovator brand that first established this specific combination of alginate and antacids for its “dual action” mechanism. While the core active ingredients (sodium alginate, sodium bicarbonate, calcium carbonate) are known, the original product possesses a proprietary formulation, encompassing specific grades and sources of alginate, precise ratios of active components, and particular excipients. These factors contribute to the unique physicochemical characteristics of its raft, such as its strength, buoyancy, and speed of formation, which have been extensively studied and clinically validated over many years. Consequently, it serves as the established reference standard for this therapeutic approach. The primary objective of this study is therefore to determine if there are significant differences in symptom control between these two formulations for patients with specific GERD symptoms who do not respond to PPI treatment. 

## Material and methods

### Study design and population

The study design was a multicenter prospective randomized controlled non-inferiority trial conducted at Srinagarind Hospital, Khon Kaen Hospital, Kalasin Hospital, and Pathum Thani Hospital between January 2023 and November 2024. All methods were carried out in accordance with relevant guidelines and regulations. The experimental protocol was approved by the Khon Kaen University Ethics Committee for Human Research based on the Declaration of Helsinki and the ICH Good Clinical Practice Guidelines (Reference No. HE651504). Informed consent was obtained from all participants prior to enrollment in the study. The study protocol was registered at the Thai Clinical Trials Registry (TCTR) on 19/01/2023 (ID TCTR20230119007).

#### Inclusion criteria

**Fig. 1:**
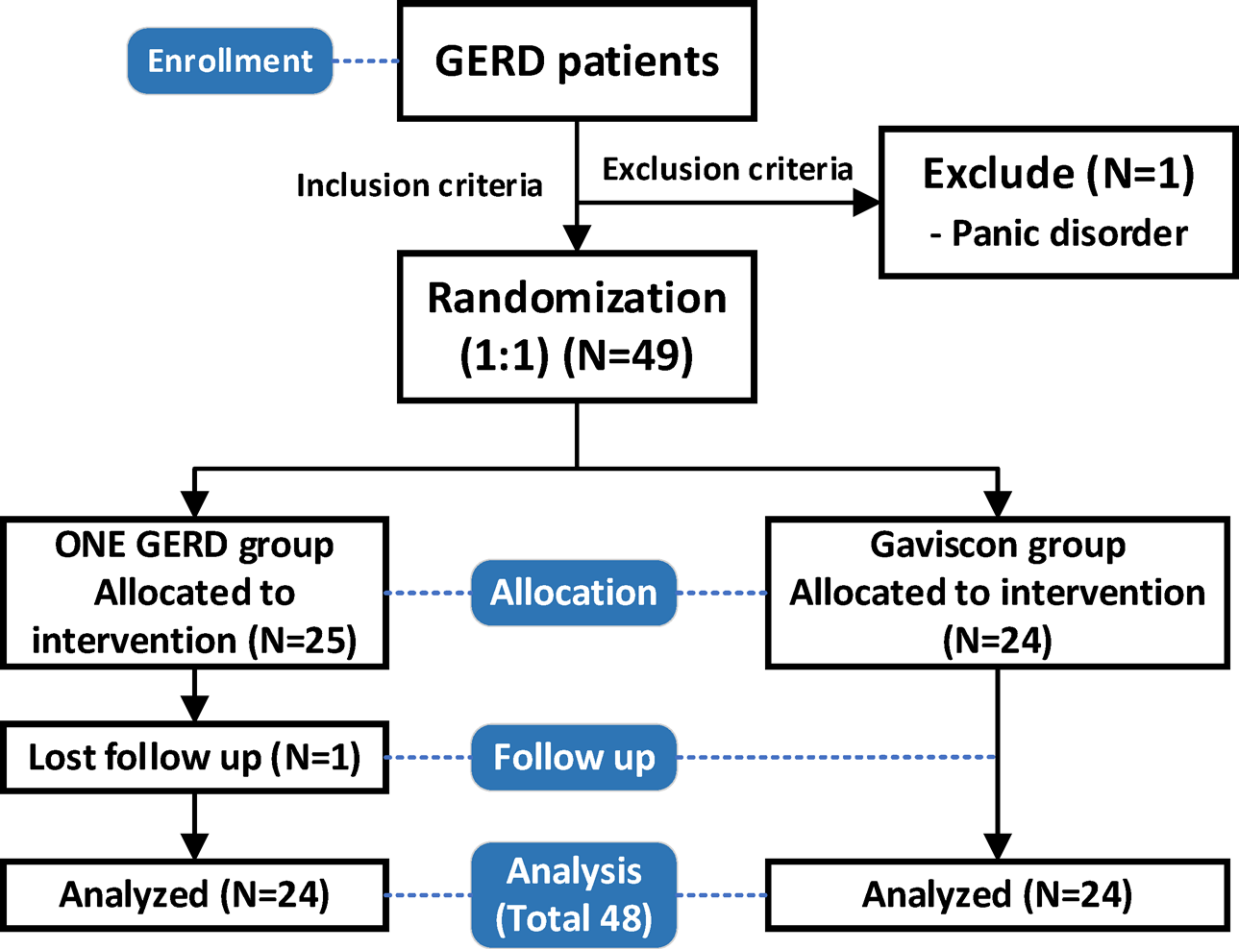
GerdQ questionnaire.

For our study involving patients were diagnosed with GERD and failed treatment with standard-dose PPIs for at least 4 weeks between January 2023–November 2024 was enrolled in the study. Inclusion criteria were patients aged 18 years and above were diagnosed with GERD by typical symptoms of GERD (Heart burn or acid regurgitation) at least 2 days/week for 1 month^5^ or ≥ 8 in Gastroesophageal Reflux Disease Questionaire (GerdQ)^7,8^. (Supplement 3 and Fig. 1), Patients presenting with alarm symptoms such as dysphagia, gastrointestinal bleeding, unexplained weight loss, frequent vomiting more than 10 times daily, and odynophagia^2^ should undergo an esophagogastroduodenoscopy (EGD) to rule out other potential diagnoses. and patients must be able to use a telephone for contact.

#### Exclusion criteria

Patients with severe psychiatric problems such as panic disorder, anxiety disorder, etc., based on DSM-5 criteria, or gastrointestinal issues such as functional dyspepsia, irritable bowel syndrome, etc., based on ROME IV criteria, and those without gastrointestinal obstruction, patients who have used prokinetic medications, histamine H2 receptor antagonists, potassium-competitive acid blockers, and alginate medications within 1 month prior to the study, patients who have taken antiplatelet medications and non-steroidal anti-inflammatory drugs (NSAIDs) within 1 week prior to the study, pregnant patients, patients who have undergone surgery on the esophagus or stomach, patients with a history of allergy to alginate medications, and patients with communication issues who cannot listen to or answer the questionnaire.

### Outcome

**Fig. 2:**
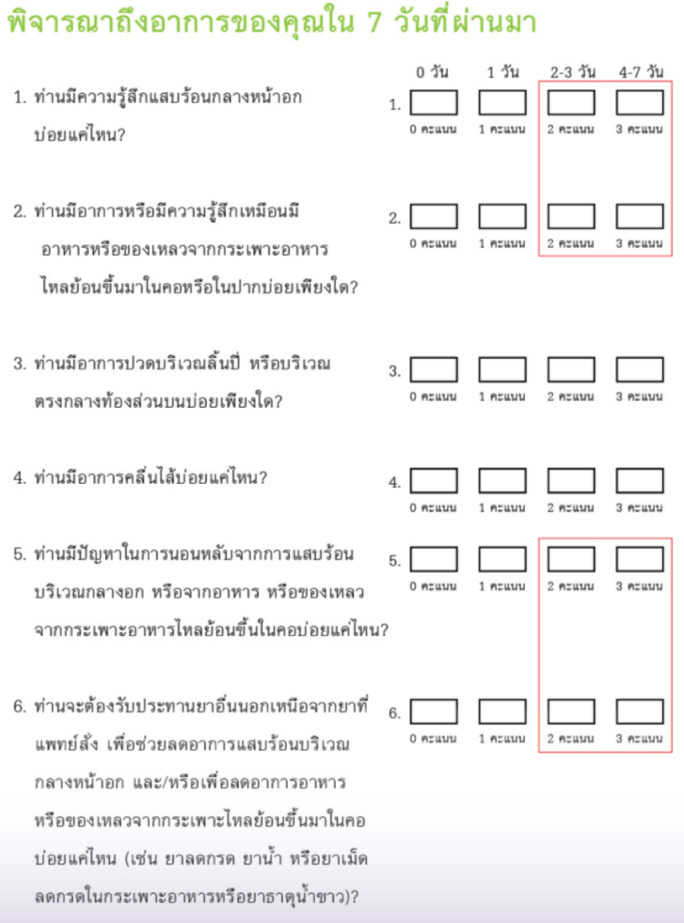
RDQ questionnaire.

The internationally standardized and globally accepted questionnaires used for diagnosing gastroesophageal reflux disease (GERD) instead of additional testing are GerdQ, while the Reflux Disease Questionnaire (RDQ) (Fig. 2) is used to evaluate symptoms after GERD treatment^5,9,10^. Both questionnaires have been widely authorized for use by their creators without copyright restrictions and can be found on general websites or in Supplement 3. The treatment response criteria specify that patients must experience symptoms no more than once per week (defined as a score of ≤ 2 points on all items/questions in Part 1 of the RDQ), with symptom severity reduced to a moderate level or lower (defined as a score of ≤ 2 points on all items/questions in Part 2 of the RDQ), as measured by the RDQ at days 7 and 28.

### Randomization

All including patients gave informed consent before study initiation. Patients were randomized to 1 of the 2 regimens at an allocation ratio of 1:1. A computer-assisted randomization process with sequences was performed using the block method (block of 2 alternating with block of 4). The statistician generated the allocation sequence and assigned the participants. The health care provider enrolled the participants. This was a double-blind study where all investigators and patients were blinded to participant allocation.

### Regimen protocol and method

The patients were randomly allocated to receive either standard-dose PPIs in combination with generic alginate (study group: ONE GERD) or original alginate (control group: Gaviscon Dual Action Suspension), taken four times daily—after meals and before bed—for 28 days. The medication packages from both experimental groups will be repackaged into identical packages, making it impossible to distinguish which type of medication is contained in each package.

The patients in both groups will receive explanations about medication administration methods and potential complications that may arise from taking the medications, such as drug allergic rashes, fainting, dyspnea, nausea, vomiting, and diarrhea. Demographic data will be collected, along with symptom assessment using GerdQ and RDQ questionnaires prior to treatment. Symptom monitoring will be conducted using the RDQ questionnaire, and medication usage will be tracked by counting remaining sachets and tablets, either by the patients themselves or their caregivers. (For smartphone users, patients or caregivers may be asked to photograph and submit evidence of remaining medication). Medication adherence must be at least 80% to be considered compliant with their assigned randomized group. A blind intermediary staff member, who is unaware of patient group assignments and must demonstrate an interpersonal correlation of no less than 90 percent, will conduct follow-up phone calls on days 7 and 28.

### Sample size calculation and statistical analyses

The sample size for this non-inferiority trial was calculated based on data from Manabe, et al.^5^. A sample size of at least 40 subjects was required to achieve 80% power at a 2-sided α of 0.05. The primary outcome was analyzed using Chi-square statistics or Fisher’s exact test with intention-to-treat analysis. A 95% confidence level was applied, and a *p*-value < 0.05 was considered statistically significant. Continuous data are expressed as means with standard deviations (SD), while categorical data are presented as numbers and percentages. All statistical analyses were performed using STATA® 10.1 software.

## Results

**Fig. 3:**
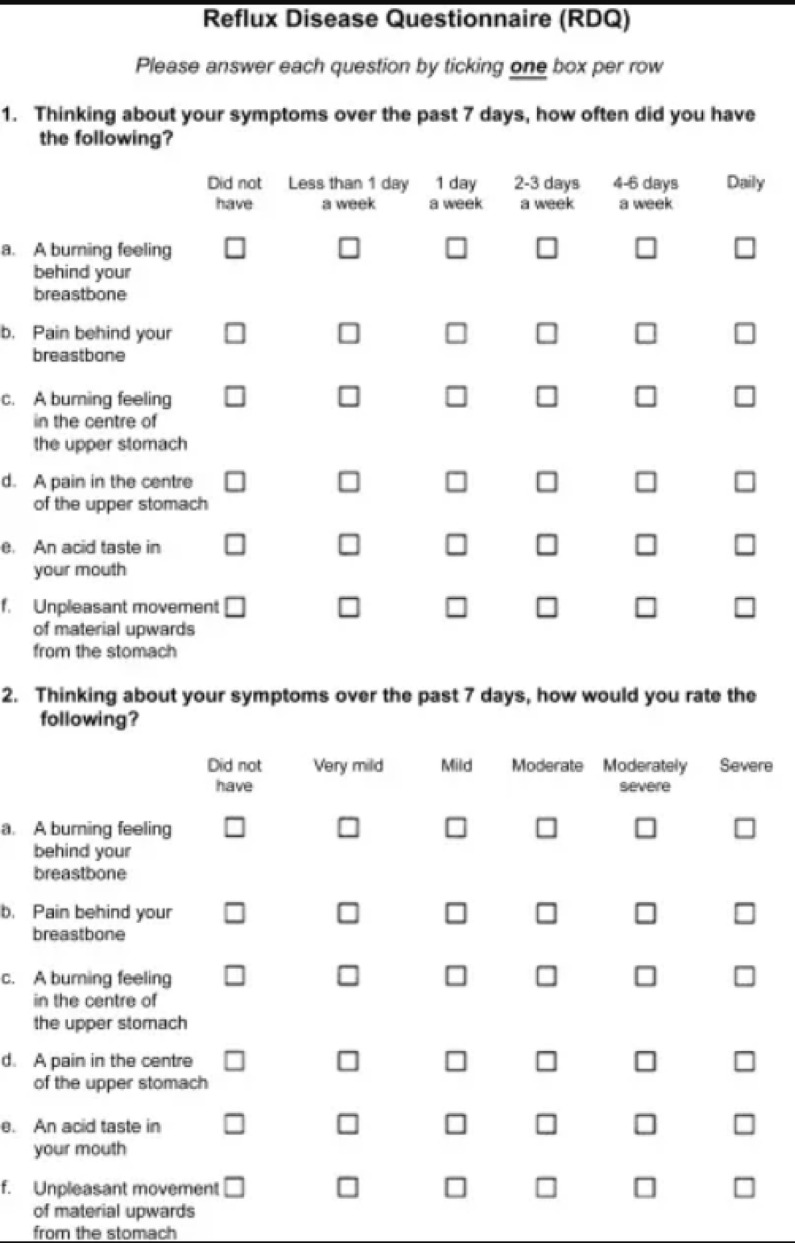
Consort diagram.

A total of 50 patients underwent screening for this study, but one patient was excluded due to panic disorder. The remaining 49 patients were successfully enrolled and randomized, with 25 patients assigned to the study group and 24 patients to the control group. One patient in the study group was lost to follow-up. Ultimately, 24 patients per group remained for analysis, as illustrated in Fig. 3.

There were no significant differences in baseline demographic characteristics between the two groups. This included the proportion of male patients (33.33% vs. 41.67%, *p* = 0.55), mean age (54 ± 13.26 vs. 53.83 ± 13.87 years, *p* = 0.97), body mass index (BMI) (23.28 ± 3.01 vs. 22.85 ± 4.11 kg/m^2^, *p* = 0.68), prevalence of underlying disease (*p* = 0.37), baseline GerdQ score (11.29 ± 2.03 vs. 10.79 ± 1.84, *p* = 0.38), and baseline RDQ score (33.16 ± 11.88 vs. 33.86 ± 12.51, *p* = 0.83), as shown in Table 1.

**Table 1:** Demographic data.

	ONE GERD (24)	Gaviscon (24)	*p* value
Male	8 (33.33)	10 (41.67)	0.55
Age	54 ± 13.26	53.83 ± 13.87	0.97
BMI	23.28 ± 3.01	22.85 ± 4.11	0.68
Underlying disease			0.37
None	14 (58.33)	17 (70.83)	
Hypertension	8 (33.33)	5 (20.83)	
Diabetes	2 (8.33)	3 (12.5)	
Dyslipidemia	6 (25)	4 (16.67)	
GerdQ score	11.29 ± 2.03	10.79 ± 1.84	0.38
Total RDQ score	33.16 ± 11.88	33.86 ± 12.51	0.83

Data are expressed as number (percentage) or mean ± SD.

At day 7 post-treatment, both groups showed identical response rates of 45.83% (*p* = 1.00), with total RDQ scores of 18.88 ± 13.92 vs. 12.71 ± 11.23 (*p* = 0.13) and symptom-free rates of 4.17% vs. 20.83% (*p* = 0.19). By day 28, response rates had increased to 54.17% vs. 70.83% (*p* = 0.23), while total RDQ scores decreased to 11.92 ± 12.00 vs. 9.08 ± 10.55 (*p* = 0.46), and symptom-free rates improved to 25% vs. 33.33% (*p* = 0.53). The overall adverse event rates showed no significant difference between groups (16.67% vs. 8.33%, *p* = 0.66), as documented in Table 2, indicating comparable safety profiles between the treatment approaches.

**Table 2:** Response rates at day 7 and day 28 after treatment with ONE GERD versus Gaviscon dual action and adverse effects.

	ONE GERD (24)	Gaviscon (24)	*p* value
D7			
Number of patients response	11 (45.83)	11 (45.83)	1.00
Total RDQ score	18.88 ± 13.92	12.71 ± 11.23	0.13
Symptom free	1 (4.17)	5 (20.83)	0.19
D28			
Number of patients response	13 (54.17)	17 (70.83)	0.23
Total RDQ score	11.92 ± 12.00	9.08 ± 10.55	0.46
Symptom free	6 (25.00)	8 (33.33)	0.53
Overall adverse event	4 (16.67)	2 (8.33)	0.66
Rash	0	1 (4.17)	1.0
Syncope	1 (4.17)	0	1.0
Dyspnea	2 (8.33)	0	0.49
Nausea/vomiting	1 (4.17)	1 (4.17)	1.0
Diarrhea	1 (4.17)	1 (4.17)	1.0

Data are expressed as number (percentage) or mean ± SD.

When analyzing patients’ symptoms, the data revealed notable findings across three key areas. For heartburn, initial patient percentages were 79.17% vs 87.5% (*p* = 0.7), which decreased to 58.33% vs 41.67% (*p* = 0.25) at day 7 and further to 37.5% vs 33.33% (*p* = 0.76) at day 28. The RDQ heartburn scores declined from baseline (5.21 ± 3.4 vs 6.0 ± 3.01, *p* = 0.4) to day 7 (3.58 ± 3.48 vs 2.33 ± 2.93, *p* = 0.17) and day 28 (1.88 ± 2.94 vs 1.33 ± 2.2, *p* = 0.58). Chest pain symptoms showed similar improvements, with initial patient percentages of 79.17% vs 75% (*p* = 0.73) decreasing to 50% vs 37.5% (*p* = 0.38) at day 7 and 25% vs 33.33% (*p* = 0.53) at day 28, while RDQ scores for chest pain decreased from baseline (5.67 ± 3.51 vs 5.46 ± 3.87, *p* = 0.85) to day 7 (2.88 ± 3.52 vs 2.25 ± 3.25, *p* = 0.41) and day 28 (1.54 ± 2.84 vs 1.46 ± 2.4, *p* = 0.74). Regurgitation symptoms followed a comparable pattern, with initial percentages of 95.83% vs 91.67% (*p* = 1) decreasing to 62.5% vs 45.83% (*p* = 0.25) at day 7 and 37.5% vs 37.5% (*p* = 1) at day 28, while RDQ scores declined from baseline (6.88 ± 2.69 vs 6.38 ± 2.84, *p* = 0.56) to day 7 (3.29 ± 3.28 vs 2.83 ± 3.52, *p* = 0.53) and day 28 (2.08 ± 3.06 vs 1.67 ± 2.55, *p* = 0.79), as documented in Table 3.

**Table 3:** Symptom-specific comparisons after treatment with ONE GERD versus Gaviscon dual action suspension.

	ONE GERD (24)	Gaviscon (24)	*p* value
D0			
Hear burn	19 (79.17)	21 (87.5)	0.7
RDQ score	5.21 ± 3.4	6 ± 3.01	0.4
Chest pain	19 (79.17)	18 (75)	0.73
RDQ score	5.67 ± 3.51	5.46 ± 3.87	0.85
Regurgitation	23 (95.83)	22 (91.67)	1.0
RDQ score	6.88 ± 2.69	6.38 ± 2.84	0.56
D7			
Hear burn	14 (58.33)	10 (41.67)	0.25
RDQ score	3.58 ± 3.48	2.33 ± 2.93	0.17
Chest pain	12 (50)	9 (37.5)	0.38
RDQ score	2.88 ± 3.52	2.25 ± 3.25	0.41
Regurgitation	15 (62.5)	11 (45.83)	0.25
RDQ score	3.29 ± 3.28	2.83 ± 3.52	0.53
D28			
Hear burn	9 (37.5)	8 (33.33)	0.76
RDQ score	1.88 ± 2.94	1.33 ± 2.2	0.58
Chest pain	6 (25)	8 (33.33)	0.53
RDQ score	1.54 ± 2.84	1.46 ± 2.4	0.74
Regurgitation	9 (37.5)	9 (37.5)	1.0
RDQ score	2.08 ± 3.06	1.67 ± 2.55	0.79

Data are expressed as number (percentage) or mean ± SD.

## Discussions

This study presents important findings regarding the comparative effectiveness of generic versus original alginates in treating GERD symptoms among patients who had previously failed standard PPI therapy. The therapeutic efficacy observed in this investigation demonstrated that both original and generic alginate formulations, when administered concurrently with PPIs, achieved identical response rates of 45.83% for symptom control in patients with GERD at day 7. These findings align with previously published data by Manabe et al.^5^, which reported a 56.7% efficacy rate for combination therapy using original alginate with PPIs. The slight variation in efficacy rates between studies may be attributed to differences in study populations, methodological approaches, or specific clinical parameters; however, the overall therapeutic trend remains consistent. The observed similarity in efficacy is likely attributable to the shared primary mechanism of action—the formation of a protective alginate raft. Both formulations are designed to create this physical barrier, which, as detailed in the introduction, relies on the interaction of alginate with gastric acid, often enhanced by co-formulated antacids.

The comparative analysis revealed no statistically significant differences in overall symptom control, RDQ scores, or specific symptoms like heartburn, regurgitation, and chest pain between the generic and original alginate groups at both 7-day and 28-day follow-ups. While research generally shows that alginate-based treatments can effectively reduce these common GERD symptoms, our findings suggest comparable effectiveness between the specific generic (ONE GERD) and original (Gaviscon Dual Action Suspension) formulations tested, with no significant advantage of one treatment over the other within the parameters of this study.

For adverse events, the similar rates between groups (16.67% vs 8.33%, *p* = 0.66) indicate that the generic alginate likely maintains a favorable safety profile comparable to the original formulation. This finding is crucial for clinical decision-making, particularly when considering cost-effectiveness.

Beyond the clinical comparability, the demonstrated therapeutic equivalence of generic alginate has significant practical implications. The lower cost typically associated with generic medications offers substantial economic benefits. For healthcare systems, particularly those operating under budgetary constraints like many in Thailand and other regions, the adoption of effective generic alternatives can lead to considerable cost savings, allowing for the reallocation of resources to other pressing healthcare needs. For individual patients, generic options reduce out-of-pocket expenses, thereby improving affordability. This enhanced affordability is a critical factor in promoting wider access to effective GERD management, especially for socioeconomically disadvantaged populations or those in areas with limited access to more expensive branded medications. Consequently, establishing the viability of generic alginates is not merely a matter of pharmacological equivalence but a step towards more equitable healthcare provision for a common and impactful condition like GERD. While a formal pharmacoeconomic analysis was outside the scope of this trial, our findings strongly support the potential for generic alginates to be a cost-effective strategy.

The study’s principal strength lies in its rigorous methodological design, incorporating multicenter participation, prospective data collection, and randomized controls. The research integrity is further enhanced by minimal attrition (only one patient lost to follow-up) and comparable baseline characteristics between study groups, establishing a robust foundation for treatment outcome comparisons.

Despite these strengths, several limitations should be acknowledged. The study’s relatively modest sample size (n = 24 per group for analysis), although calculated to achieve 80% power for the primary outcome based on existing data for non-inferiority, may have restricted the statistical power to detect smaller, yet potentially clinically meaningful, differences between the generic and original alginates across all secondary outcomes or subgroup analyses. Given that most of the observed differences between the two groups were not statistically significant (*p* > 0.05), there is an inherent risk of a type II error, where a true, albeit potentially small, difference between the treatments might exist but was not identified due to insufficient sample size. This is particularly relevant when considering that subtle variations in alginate source, grade, or the precise quantities and types of excipients as discussed in the introduction regarding potential differences in raft characteristics could lead to nuanced variations in efficacy or tolerability that a larger study might uncover. Furthermore, a significant limitation is the 28-day follow-up period. Given the chronic nature of GERD, this duration may be too short to fully assess the complexities of long-term symptom management and the sustained safety and efficacy of these treatments. While providing valuable initial insights, it precluded a comprehensive evaluation of sustained symptom control beyond this acute phase. Importantly, longer follow-up periods are crucial to investigate other clinically relevant outcomes such as potential esophageal mucosal healing (which could be assessed via endoscopy), to objectively measure any changes in esophageal acid exposure (e.g., through ambulatory pH monitoring or impedance-pH monitoring), and to identify any potential long-term or cumulative adverse effects that might not manifest within a four-week trial. Addressing these aspects through studies with extended observation periods would significantly improve the clinical relevance of the findings for managing a chronic condition like GERD and provide a more complete understanding of the comparative long-term utility of generic and original alginate formulations.

To strengthen the evidence base, more definitively confirm the findings of therapeutic equivalence or non-inferiority, and enhance the rigor and mechanistic understanding of treatment effects, future research would benefit not only from larger, adequately powered cohorts and extended observation periods but also by incorporating objective physiological or biomarker assessments. Such assessments could include esophageal pH monitoring to directly quantify acid exposure, esophageal manometry to evaluate motor function and lower esophageal sphincter integrity, or endoscopic evaluation with biopsies for markers of mucosal inflammation. Combining symptom questionnaires with these objective measures would provide a more comprehensive evaluation of treatment efficacy and deepen insights into the pathophysiological response to generic versus original alginate therapies, potentially revealing subtle differences in their impact on esophageal physiology beyond subjective symptom relief.

## Conclusion

This study provides evidence supporting the therapeutic equivalence of the generic alginate (ONE GERD) to the original formulation (Gaviscon Dual Action Suspension) in treating symptoms for patients with GERD who have failed PPI therapy. Crucially, the comparable efficacy and safety, coupled with the inherent lower cost of generic medications, suggest significant economic benefits and the potential for wider patient access to effective GERD management. This makes generic alginate a viable and attractive alternative in clinical practice, particularly in resource-limited settings or for patients facing financial constraints, thereby contributing to more equitable healthcare solutions without compromising therapeutic outcomes.

## Supplementary Information

Supplementary Information.

## Data Availability

The datasets used and/or analyzed during the current study are available from the corresponding author upon reasonable request.
