# Supplementary material for: Comparable efficacy of generic and original alginate for symptom control in PPI-refractory GERD
Source: Sci Rep. 2025 Aug 4;15:28429. doi: 10.1038/s41598-025-13400-w (PMC12322105; doi:10.1038/s41598-025-13400-w)
Supplement: Supplementary file 1 — Supplementary Information. [file 41598_2025_13400_MOESM1_ESM.pdf]

Supplement 1

ONE GERD is a trade name for a generic medication containing Dual Action Sodium Alginate.

Indicated for:

1. Relief of heartburn with acid reflux and indigestion caused by acid reflux
2. Relief of heartburn symptoms, acid reflux, indigestion due to acid reflux, and reduction of dyspepsia.

The medication comes in a suspension form packaged in 10 ml sachets. Each sachet contains the following active ingredients:

1. Sodium alginate 500 mg
2. Sodium bicarbonate 213 mg
3. Calcium carbonate 325 mg

Dosage and Administration: For adults and children 12 years and older: Take 10-20 milliliters after each of the four main meals.

Supplement 2

Gaviscon Dual Action Suspension is a trade name for a generic medication containing Dual Action Sodium Alginate.

Indicated for:

3. Relief of heartburn with acid reflux and indigestion caused by acid reflux
4. Relief of heartburn symptoms, acid reflux, indigestion due to acid reflux, and reduction of dyspepsia.

The medication comes in a suspension form packaged in 10 ml sachets. Each sachet contains the following active ingredients:

4. Sodium alginate 500 mg
5. Sodium bicarbonate 213 mg
6. Calcium carbonate 325 mg

Dosage and Administration: For adults and children 12 years and older: Take 10-20 milliliters after each of the four main meals.

Supplement 3

The Gastroesophageal Reflux Disease Questionnaire (GerdQ) (Figure 2) is a diagnostic tool used to help determine whether a patient has GERD. This questionnaire relies solely on symptoms and does not require an endoscopy or additional tests for diagnosis. A total score of 8 or higher on the GredQ indicates a diagnosis of GERD.

The Reflux Disease Questionnaire (RDQ) (Figure 3) is a diagnostic assessment tool used to evaluate the treatment response to medication in patients with GERD. The questionnaire is administered at two time points: before initiating treatment and seven days after treatment begins. A reduction in symptom scores between these two assessments indicates a positive response to the prescribed medication.
